# Thermal and Mechanical Characterization of the New Functional Composites Used for 3D Printing of Static Mixers

**DOI:** 10.3390/ma15196713

**Published:** 2022-09-27

**Authors:** Marijan-Pere Marković, Ivan Karlo Cingesar, Laura Keran, Domagoj Prlić, Ivana Grčić, Domagoj Vrsaljko

**Affiliations:** 1 Faculty of Chemical Engineering and Technology, University of Zagreb, Marulićev Trg 19, 10000 Zagreb, Croatia; 2 Faculty of Geotechnical Engineering, University of Zagreb, Hallerova Aleja 7, 42000 Varaždin, Croatia

**Keywords:** additive manufacturing, fused filament fabrication, photocatalysis, composites, static mixers

## Abstract

This paper investigates the possibility of integrating the combination of nanofillers, titanium dioxide (TiO_2_) and carbon nanotubes (CNT) into the thermoplastic polymer matrix. This combination of fillers can possibly modify the physico-chemical properties of composites compared to the pure polymer matrix. The composites were blended using the extrusion method. The composite filament produced was used to manufacture static mixers on a 3D printer using the additive manufacturing technology fused filament fabrication (FFF). The aim of this work was to inspect the influence of the filler addition on the thermal and mechanical properties of glycol-modified polyethylene terephthalate (PET-G) polymer composites. The fillers were added to the PET-G polymer matrix in several ratios. Tensile test results showed an increase in the overall strength and decrease in the elongation at break of the material. Melt flow rate (MFR) showed a decrease in the viscosity with the initial filler addition and reaching a plateau after 2 wt% filler was added. Differential scanning calorimetry (DSC) and thermogravimetric analysis (TGA) showed minor changes in the thermal properties. Scanning electron microscope (SEM) results showed homogenous distribution of the filler in the matrix and strong filler–matrix adhesion. The results indicate suitable properties of new functional composites for the 3D printing of static mixers for application in tubular reactors.

## 1. Introduction

Additive manufacturing (AM) is a process in which 3D objects are built layer by layer from a 3D digital model [1]; it is commonly known as 3D printing. The manufacturing process begins with the creation of the 3D model usually using computer-aided design (CAD) software [2,3,4] or by scanning the existing object using 3D scanners [5]. While the scanners limit the 3D model to the external appearance of the existing object, the CAD software offers a much wider range of possibilities, such as relatively easy modification of the model and adaptation of both the external and internal shape and structure of the object to the application and manufacturing requirements [5]. With the rapid progress of additive manufacturing [6,7], the accompanying software is also evolving, developing and becoming more user-friendly and easier to use. Thus, it is possible to choose any from the wide range of CAD software (e.g., FreeCAD, AutoCAD, Catia, Solidworks, Blender, and Autodesk Fusion 360). This manufacturing method offers the possibility of creating highly complex geometries [8,9] with high quality and accuracy at an affordable price [10]. This is not possible with conventional methods.

One of the most well-known and widely used additive manufacturing technologies is fused filament fabrication (FFF) [5]. This technology is also often termed as fused deposition modelling (FDM) [11]. The 3D printer’s main parts are an extruder and a heated bed. FFF uses thermoplastic polymer materials in the form of a 1.75mm- or 3mm-diameter filament. The material is extruded through the nozzle onto the heated bed layer by layer [5,12].

Before the actual 3D printing, the model created in the CAD software must be translated into code that the 3D printer can process. This is conducted in the so-called slicer software. In the slicer software, print settings are defined, such as the nozzle temperature, the bed temperature, the print speed, the cooling fan speed, the infill shape and density, and many more. In addition, the support structures required for successful prints are defined and created for the overhanging parts of the model [12].

Polymer matrices offer a cost-effective option for materials that have a wide range of capabilities regarding physico-chemical properties and configuration options. It is also relatively easy to modify the properties of polymer materials by making composites, i.e., by the addition of the fillers, which gives them a much broader range of applications. Fillers can be added to the polymer matrix in various forms and contents. Due to their effects on the properties of the composite material, it is important to achieve good mixing and homogeneity [13,14,15,16,17]. The addition of fillers, especially more than 10 wt%, poses a number of problems for this technology when 3D printing the composite material. Surface roughness, low detail quality, and nozzle clogging are some of the most common [18]. For this reason, other 3D-printing technologies such as SLA (stereolithography) and DLP (digital light processing) are often used for composites containing higher filler content (>10 wt%) [19,20]. One of the well-known fillers is titanium dioxide (TiO_2_).

Carbon nanotubes (CNTs) are an allotropic modification of carbon that have a cylindrical nanostructure. They possess a variety of advantageous properties such as low mass, high electrical and thermal conductivity, excellent mechanical properties, etc. [21], but have no photocatalytic properties. CNTs have been shown to enhance the photocatalytic activity of TiO_2_ due to a drastic synergistic effect as described in the work of Ahmmad et al. [22]. The photocatalytic effect, which can be enhanced by using the CNTs as electron transfer channels, induces the formation of the highly reactive radical ions from adsorbed oxygen [23].

During the preparation of composite materials, it is necessary to monitor the changes in mechanical and thermal properties in order to determine the 3D printability and end-use applicability of the material. Mechanical properties describe the behavior of materials under the influence of stress or deformation under an applied force. Material structure and composition affect mechanical properties, as do temperature, load duration, and force direction [24]. The thermal properties of the polymer are defined by three major thermal transitions: glass transition, melting, and crystallization. These transitions can give an indication of the compatibility of the phases within the composite and the thermal stability of the material [25].

The aim of this work is to prepare functional PET-G/TiO_2_ and PET-G/TiO_2_/CNT filaments with different filler contents and to evaluate the possibility of 3D printing static mixers with the produced composites. The composites will be studied in order to determine the influence of the filler addition on the thermo-mechanical properties of the material. The photocatalytic properties of these composites will be tested in systems for the purification of polluted water using sunlight as a radiation source in subsequent research. These composites offer a novelty in the field of polluted-water purification in comparison with previous works of 3D printing monoliths containing TiO_2_ by paste extrusion technology [26] or by creating TiO_2_ films on the polymer surface [27,28,29]. Additionally, 3D printing of the photocatalytic composite material allows the fabrication of new static mixers with complex geometries.

## 2. Experimental Section

### 2.1. Materials

In this work, glycol-modified polyethylene terephthalate (PET-G) was used as the polymer matrix (Devil Design, Mikolow, Poland). Nano-titanium dioxide, trade name AEROXIDE TiO_2_ P25 (Evonik industries, Essen, Germany), and multi-walled carbon nanotubes (MWCNT) (Sigma Aldrich, Burlington, MA, USA) were used as fillers for the production of functional composite filaments. The manufacturer of the PET-G recommends using the temperature range of 220 °C to 250 °C for the nozzle and the range of 70 °C to 80 °C for the platform during the 3D printing. The manufacturer data for TiO_2_ indicate the mass ratio of anatase:rutile = 80:20, bulk density of nanoparticles equal to 130 g/L, a specific surface area of 50 m^2^/g and the particle size range of 10 to 50 nm. The CNTs are specified as multi-walled (MWCNT), with 50 to 90 nm diameter and >95% carbon basis.

### 2.2. Sample Preparation

The purchased PET-G filament was in the shape of filament with a 1.75 mm diameter and was first cut into granules on a Rondol granulator. These pure PET-G granules were then dry blended for 2 min with varying TiO_2_ and CNT filler contents (Table 1).

The obtained mixtures were then melted and blended in a Rondol 21 mm LAB TWIN twin-screw extruder with the temperature zones indicated in (Table 2) at a speed of 50 rpm. The samples with higher filler content were extruded at higher temperature due to their higher viscosity. The extruded composite filaments were cooled in a distilled water bath and, after cooling, were again cut into granules on the granulator. No particular challenges were encountered in the fabrication process, such as polymer burnout or nozzle clogging due to filler agglomeration.

The composite granules were fed into a Noztek Pro single-screw extruder for the production of the filament. The diameter of the extruded filament was 1.75 mm ±5%, which is required for the 3D printer. All filaments were extruded at 186 °C and air-cooled with the built-in ventilator. This extruder has a single rotation speed. The extruded functional filaments were later used in a 3D printer to produce functional static mixers.

The samples for the tensile test were prepared on the Fontijne hydraulic press. The composite granules were placed in a steel mold with dimensions 100 mm × 10 mm × 1 mm and covered with Teflon sheets to prevent sticking. The molds containing the composites were first preheated at 190 °C for 4 min, then pressed at 15 MPa for 4 min and finally cooled for 4 min before the mold was removed. The pressed plates were cut into the desired shape using a tensile test sample punch. The punch used was manufactured according to the standard DIN EN ISO 8256, specimen type 3 [30]. The samples for the tensile test were molded instead of 3D printed in order to determine the mechanical properties of the material itself without the influence of 3D-printing parameters.

### 2.3. Characterization

The functional composites were characterized using the Davenport melt flow rate (MFR) tester. The viscosity of the composite is inversely proportional to the melt flow rate. An amount of 6–8 g of composite granules was placed in the machine at a working temperature of 190 °C, and the press weight used was 5 kg. The results were obtained as the mass of sample that flowed out of the machine within 10 min, in grams. The MFR value was calculated according to the equation:

$$\mathrm{MFR}=\left(\frac{600}{t}\right)\times m$$

where *t* is the flow time of the sample in seconds and *m* is the mass of the sample which flowed through the tester in grams, weighted on the analytical scale.

Differential scanning calorimetry (DSC) was used to determine the glass transition temperature shift upon addition of filler content using a Mettler Toledo 823^e^ measuring module. The analysis was conducted in two heating cycles with a nitrogen flow of 50 mL/min as inert atmosphere.

First cycle:
Heating 0–260 °C at 10 °C/min.
Isothermal 260 °C for 2 min.
Cooling 260–0 °C at −10 °C/min.
Isothermal 0 °C for 2 min.
Second cycle:
Heating 0–260 °C at 10 °C/min.
Cooling 260–25 °C at −30 °C/min.

Thermogravimetric analysis (TGA) was performed on a TA Instruments Q500 instrument to determine the thermal stability of the materials. The onset of weight loss was monitored as the initial decomposition temperature. The tests were performed in the temperature range of 25–500 °C at 10 °C/min in a nitrogen flow of 50 mL/min as an inert atmosphere.

The mechanical properties of the prepared samples were determined with a tensile test on a Zwick Röell AG UTM 1445 universal testing machine. The crosshead speed used was 10 mm/min and the initial gauge length was 30 mm.

The surface of the composites was examined using a scanning electron microscope (SEM) Tescan Vega 3 SEM. Samples for the microscope were first sputter coated with conductive platinum/palladium and then examined in SEM with a secondary electron (SE) detector.

### 2.4. 3D Printing

**Figure 1:**
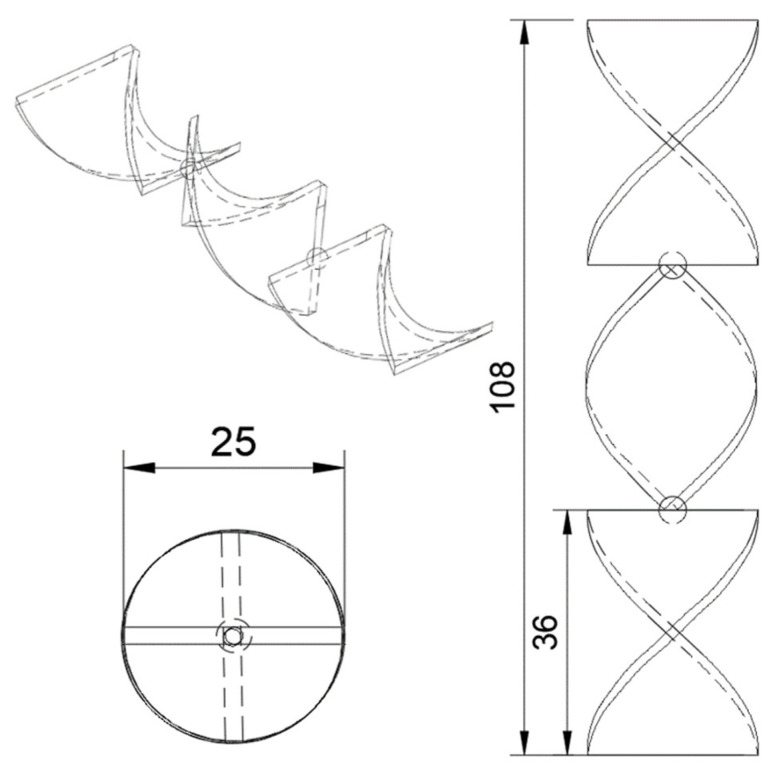
Static mixer dimensions in millimeters.

**Figure 2:**
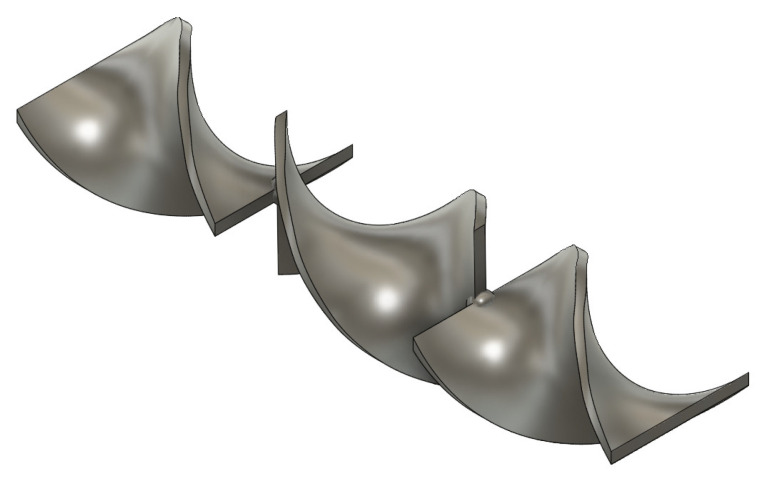
Static mixer model from Autodesk Fusion 360 CAD software.

**Figure 3:**
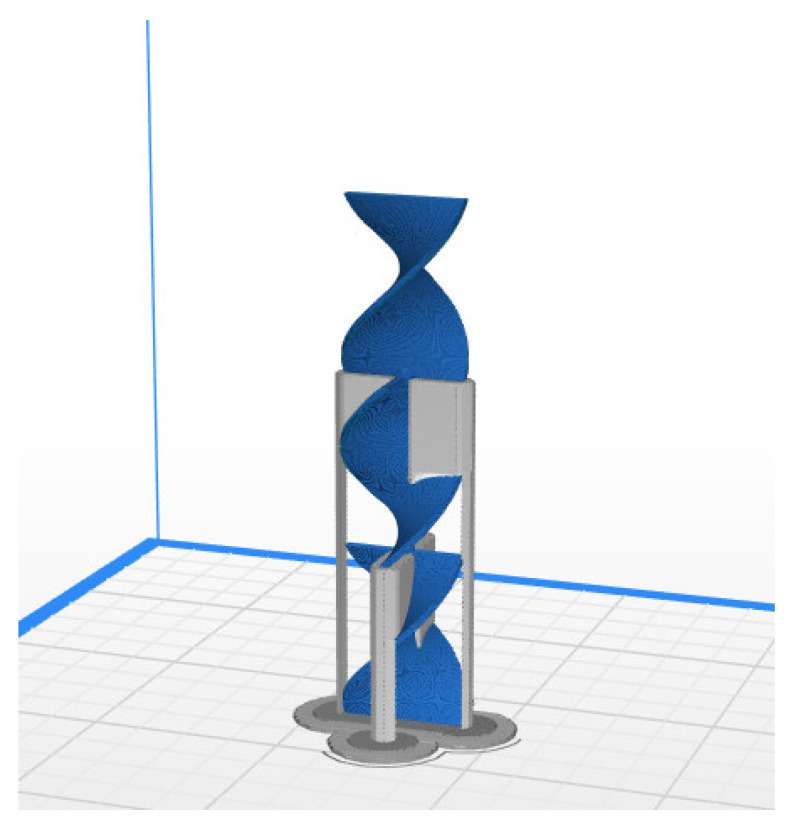
Static mixer model with defined print settings and support structures from Z-suite slicer software.

In this work, a Zortrax M200 3D printer was used. The printer uses fused filament fabrication (FFF) additive manufacturing technology. As aforementioned, the filament for this printer is required to have a diameter of 1.75 mm ±5%. The first step of the process is to design a 3D model of the static mixer [31] using Autodesk Fusion 360 CAD (Computer-Aided Design) software (Figure 1 and Figure 2). The designed model is later exported in the .stl format. This format is used in the slicer program Z-suite, where the print settings are defined and the model is transferred to the 3D printer (Figure 3).

## 3. Results and Discussion

### 3.1. Prepared Composite Filaments

**Figure 4:**
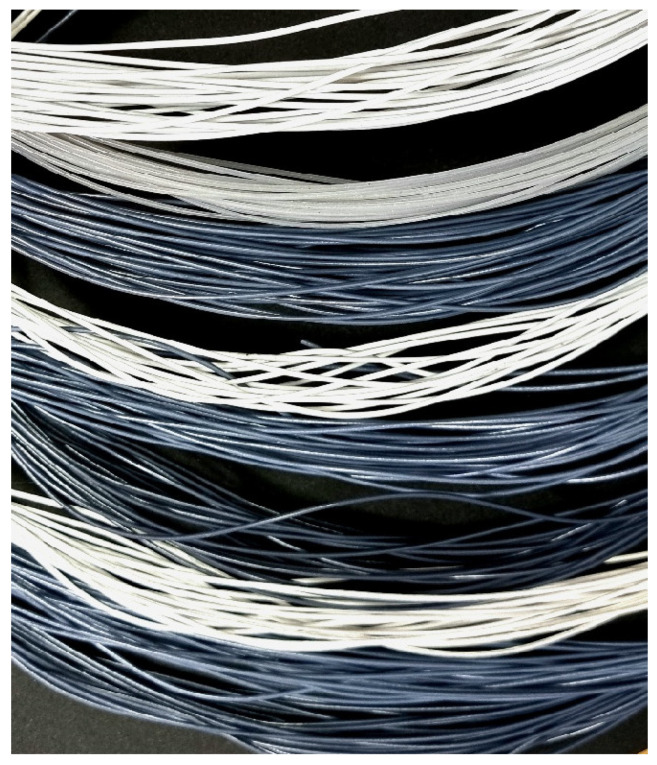
Extruded composite filaments for 3D printing with increasing filler content from top to bottom: PET-G 1.5T 0C, PET-G 1.5T 0.25C, PET-G 1.5T 0.5C, PET-G 3T 0C, PET-G 3T 0.25C, PET-G 3T 0.5C, PET-G 6T 0C, PET-G 6T 0.25C, and PET-G 6T 0.5C.

The commercially available PET-G and the prepared and extruded composite filaments with different filler contents are shown on Figure 4. The diameters of the produced filaments were within 1.75 mm ±5%, as required for the 3D printing. The visual appearance of the prepared filaments indicates good homogeneity of the samples.

### 3.2. MFR Analysis

The MFR was studied to determine whether the viscosity of the composites changed with the increase in the added filler content. The results obtained are shown in Table 3. For the first four samples, a decreasing trend in the viscosity of the samples with the increase in the filler content could be seen as the MFR value increased. The samples PET-G 3T 0C and PET-G 3T 0.25C showed a significant deviation from this trend. This deviation is best explained as an artefact caused by the inhomogeneity of the filler distribution in the matrix, and by a poorly chosen representative sample due to the very small test sample mass. Excluding the two deviating samples, it can be seen that the viscosity reached an overall plateau at ≥2% mass of the added filler content combined. Such results are in agreement with the work of Seong-Hun et al. [32], who analyzed the MFR of pure PET-G compared to PET-G/TiO_2_ composite and found no significant difference between the values of the samples. Car et al. [33] determined the MFR of Z-GLASS material (based on PET-G) and its composite with TiO_2_ filler and found an increase in the MFR value with increasing filler content, similar to the MFR value increase with the lower filler content of this work. This increase in the MFR values or decrease in the viscosity is not necessarily filler-dependent, but can be explained by the theory of polymer chain length scission due to degradation during thermal processing, as shown by Colin et al. [34].

### 3.3. DSC Analysis

**Figure 5:**
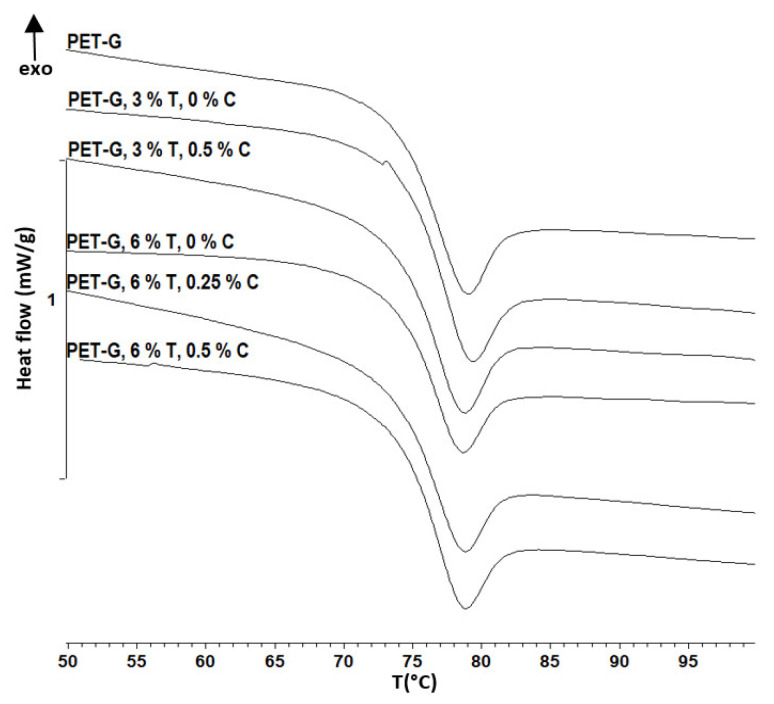
Graphical representation of DSC test results of PET-G and composite samples.

DSC has been used as a standard method for determining thermal properties. Composite materials are often analyzed using this method to monitor possible shifts in thermal phase transitions. In this work, the glass transition temperature was monitored by comparing the pure PET-G polymer with the prepared composites (Figure 5). From the results given in Table 4, there was no significant shift in glass transition temperature. For all the samples, the determined glass transition temperature was within the range of 2 °C. Similar results were obtained by Wang et al. [35] and El Fray et al. [36] in their studies regarding PET-G and PET composite matrixes with the addition of TiO_2_ filler, indicating that TiO_2_ as a filler does not affect this property of the PET-G polymer.

### 3.4. TGA Analysis

**Figure 6:**
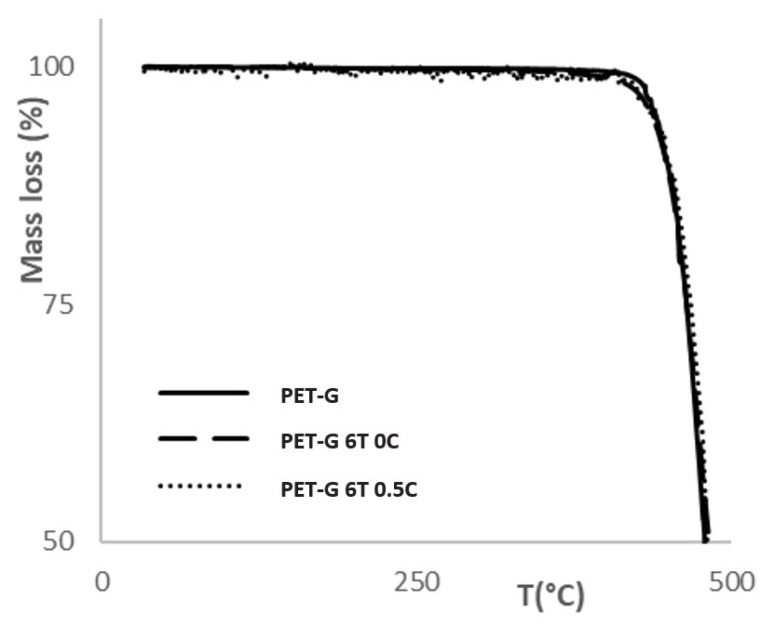
Graphical representation of TGA test results of PET-G and composite samples.

TGA analysis was performed to determine the thermal stability of the materials. This property defines the working range and applicability of the material at elevated temperatures. The results of the thermogravimetric analysis are shown in Table 5. It can be seen that the addition of fillers did not affect the thermal stability of the composite materials compared to pure PET-G, as all of the samples showed a thermal decomposition temperature within ± 2 °C. All tested materials showed a mass loss of less than 3% up to 400 °C (Figure 6). Wu et al. [37] reported similar thermal stability results with PES/TiO_2_ composite; they noticed a slight increase in the decomposition temperature with the increase in the filler content, but added only up to 0.7 wt% filler. Wang et al. [38] investigated MWCNT/TiO_2_ composite and found similar results to the ones presented in this work. They reported no significant mass loss during 2 h at 400 °C. These results make the prepared composite material eligible for use in photocatalytic purification at elevated temperatures.

### 3.5. Tensile Test

**Figure 7:**
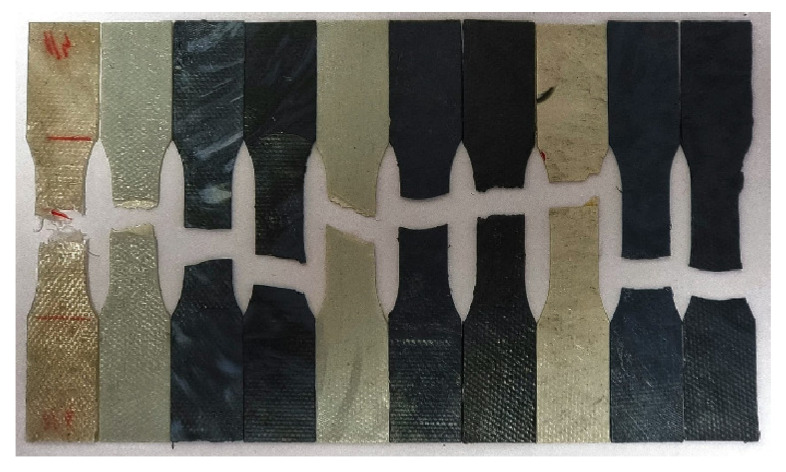
Test samples in order as in Table 6 after tensile testing.

**Figure 8:**
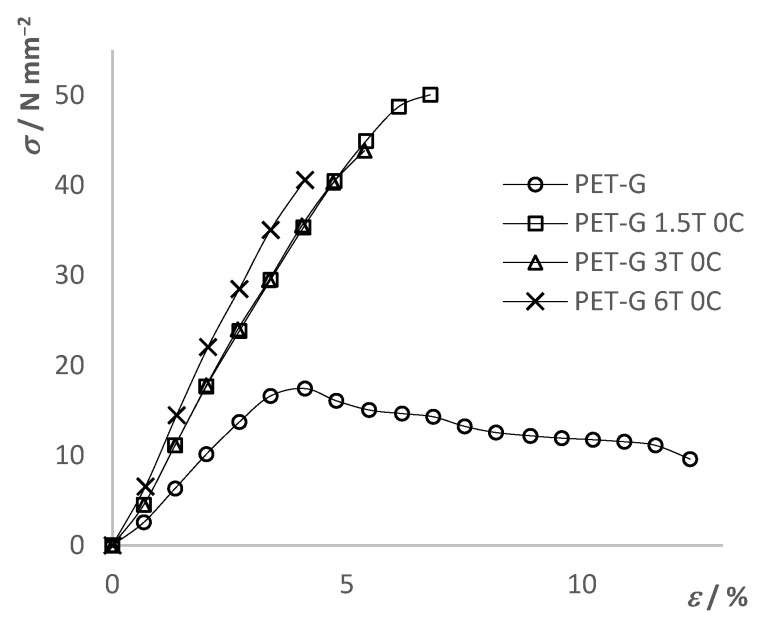
Tensile test results of PET-G and PET-G/TiO_2_ composites.

**Figure 9:**
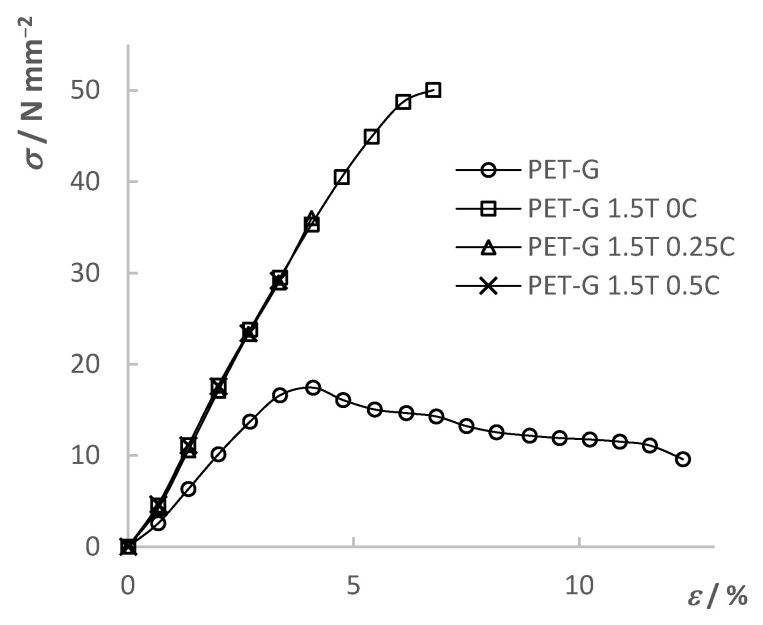
Tensile test results of PET-G and PET-G/1.5% TiO_2_ composites with added CNT ratios.

**Figure 10:**
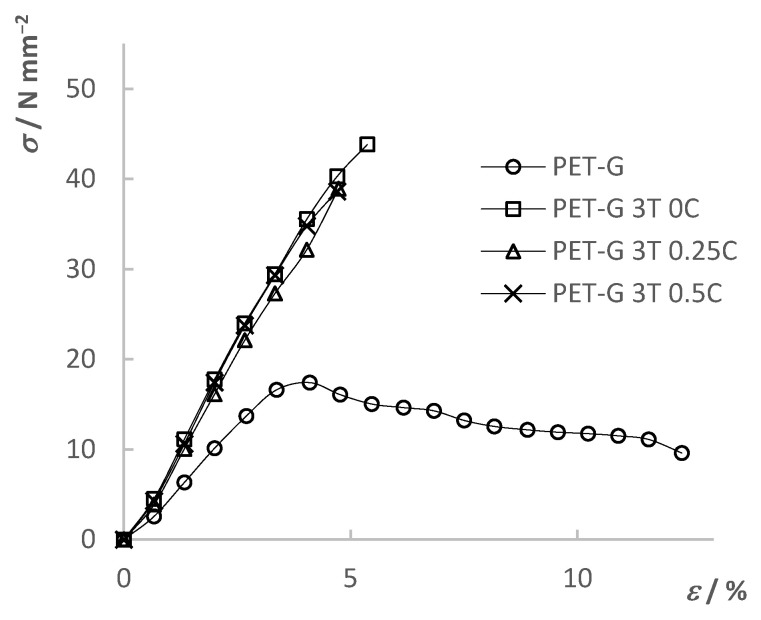
Tensile test results of PET-G and PET-G/3% TiO_2_ composites with added CNT ratios.

**Figure 11:**
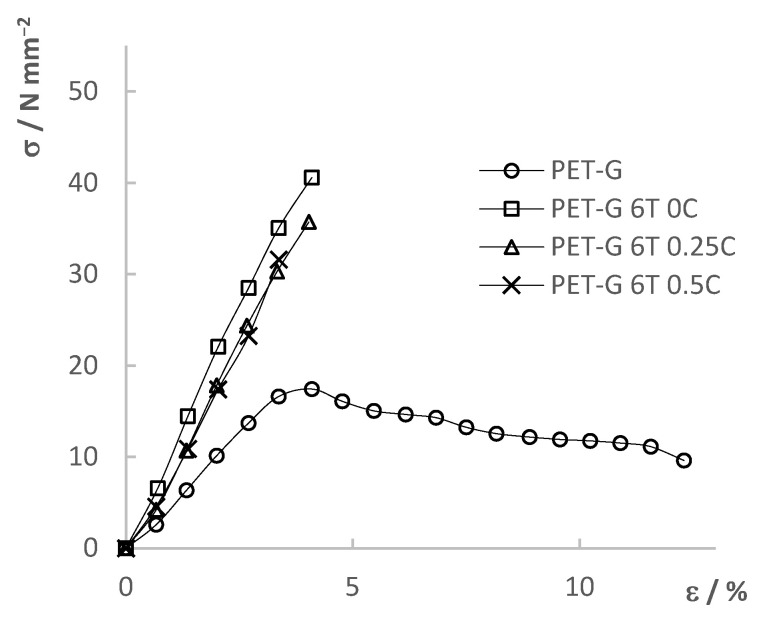
Tensile test results of PET-G and PET-G/6% TiO_2_ composites with added CNT ratios.

The tensile mechanical properties of the composites (Figure 7) were determined using a universal testing machine (UTM). As a result, stress–strain curves were obtained. The determined mechanical properties were compared between the composites themselves depending on the added filler content as well as with the results of the pure PET-G. The results are shown in Figure 8, Figure 9, Figure 10 and Figure 11 and summarized in Table 6.

Figure 8 shows the results of the tensile test for the PET-G composite material with different TiO_2_ filler content. From the results, it can be seen that the initial addition of TiO_2_ significantly changed the mechanical properties of the composite material compared to pure PET-G. The tensile strength of the composite increased by 233%–288%, while the maximum elongation decreased from 12.3% to a range of 3.3%–6.8%, depending on the filler content. Moreover, the composite materials showed a brittle fracture while PET-G was a tough material. Additionally, it can be seen that the differences in the mechanical properties in between composite materials with further addition of the filler content were smaller compared to differences between composites and pure PET-G. The trend of decreasing tensile strength and elongation at break can be seen with a further increase in the TiO_2_ filler content. By the slope of the linear part of the curves, it can be seen that Young’s modulus increased with the addition of the filler, indicating an increase in the stiffness of the material.

Figure 9, Figure 10 and Figure 11 show the tensile test results of the composites with different CNT filler content added to the respective TiO_2_ filler content. Each stress–strain diagram includes the PET-G curve, which is to be seen as a reference line. A trend towards decreasing tensile strength and elongation at break with increasing filler content can be seen. Addition of the fillers, especially in larger quantities, can cause steric hindrance within the material depending on the compatibility of the composite phases. Such an effect causes lower mobility of the polymer chains; hence, it causes far lower elongation at break in the case of the composites. Additionally, addition of the fillers in the polymer matrix can lead to physical crosslinking. Created bonds between the polymer molecules can lead to both greater strength of the composite and lower elongation due to the lower mobility of the chains. An increase in the filler content in the polymer matrix can lead to the development of internal stresses within the composite structure. Additionally, possible inhomogeneity and possibly trapped air bubbles in the material are all locations of stress concentration which can cause premature yield of the composite material. The work by Car et al. [33] mentions problems with inhomogeneity and entrapped air bubbles when TiO_2_ filler is mixed into the polymer (Z-GLASS, PET-G-based material) matrix and preforming a tensile test. These problems greatly affected the results, but ultimately, the conclusion was also that the further increase in filler content led to the decrease in mechanical properties. Increasing the filler content can affect the mechanical properties especially if the interfacial compatibility is poor, which can lead to adhesion problems between the matrix and filler. Such defects in the internal structure lead to crack formation which progresses rapidly under stress, as explained in the work of Sathish Kumar et al. [39] using the example of a PET-G matrix with a PGA dispersed phase.

The results show that the fillers have a great influence on the mechanical properties of the material, but also that all the results obtained are compliant with the objective of the final application, which is the 3D printing of static mixers by using the prepared composites. The molded samples displayed the mechanical properties of the material solely compared to the 3D-printed test samples, which can show the great influence of the 3D-printing parameters, such as model orientation, print direction, layer thickness, etc., on the mechanical properties [40].

### 3.6. SEM

SEM was used to examine the fracture surface of the samples after the tensile test and the surface of the layers of the static mixers after 3D printing. The samples for SEM examination were selected in the case of tensile test samples: the sample with the highest TiO_2_ filler content (PET-G 6T 0C) and the sample with the highest content of the both TiO_2_ and CNT fillers (PET-G 6T 0.5C); and in the case of 3D-printed static mixers: samples PET-G 1.5T 0.25C and PET-G 3T 0C, since they presented the most difficulties during 3D printing.

**Figure 12:**
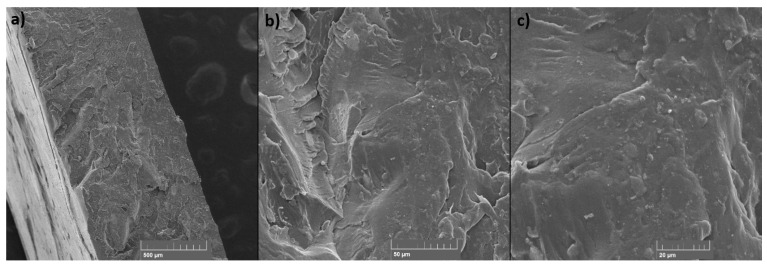
SEM micrographs of the fracture surface for the PET-G 6T 0C sample at (**a**) 100×, (**b**) 1000× and (**c**) 5000× magnification.

**Figure 13:**
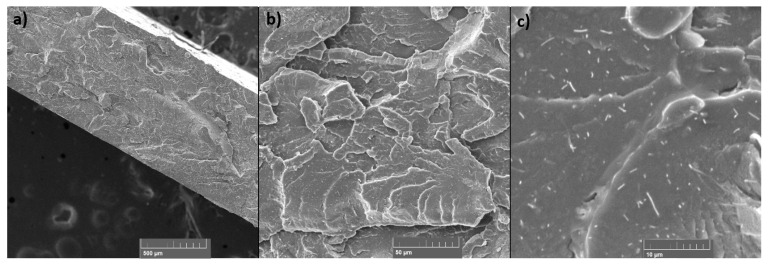
SEM micrographs of the fracture surface for the PET-G 6T 0.5C sample at (**a**) 100×, (**b**) 1000× and (**c**) 2000× magnification.

SEM micrographs presented in Figure 12 and Figure 13 show the fracture surfaces of the specimens after the tensile test. A good homogeneity of the TiO_2_ can be seen in the sample with the highest filler content (Figure 12). Micrographs show a good distribution of the filler and no phase separation. The particle size of the filler is <1 µm, as can be seen. The sample with CNT filler (Figure 13) also shows good homogeneity and filler distribution and no phase segregation. The size of the CNTs ranges from 0.1 µm to 4 µm in length and up to 0.1 µm in width, as can be seen in the micrographs with the highest magnification. No visible agglomeration of the filler particles can be seen in either sample.

**Figure 14:**
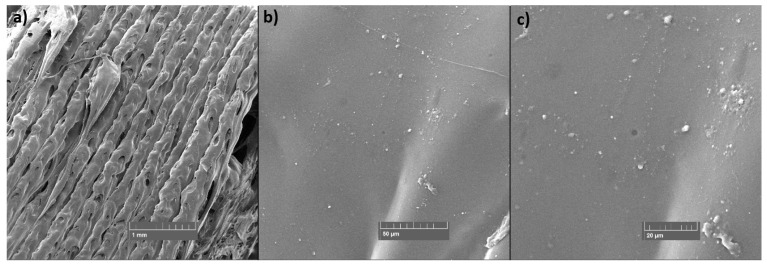
SEM micrographs of the 3D-printed static mixer for the PET-G 1.5T 0.25C sample at (**a**) 50×, (**b**) 1000× and (**c**) 2000× magnification.

**Figure 15:**
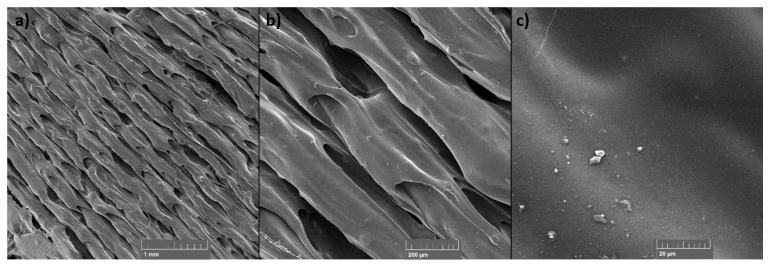
SEM micrographs of the 3D-printed static mixer for the PET-G 3T 0C sample at (**a**) 50×, (**b**) 200× and (**c**) 2000× magnification.

**Figure 16:**
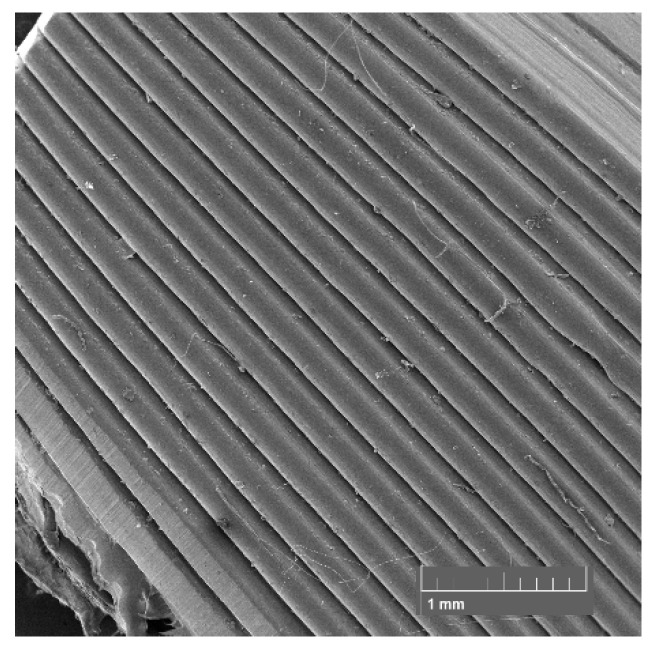
SEM micrograph of the 3D-printed static mixer from pure PET-G at 50× magnification.

The SEM micrographs presented in Figure 14 and Figure 15 show the surface of the static mixers 3D printed from composites. With both samples, imperfections in layer placement and roughness of the surface can be seen compared to the layers of the pure PET-G (Figure 16). In this specific application, such roughness may have a beneficial effect, as it increases specific surface area for the catalyst to work. At the higher magnifications (1000× and 2000×), particles of the filler can be seen on the surface of the 3D-printed static mixers. Good distribution with the particle size range from 0.1 µm to 4 µm is visible. Additionally, some agglomeration of the TiO_2_ can be seen at the micrograph of the sample containing CNT filler (Figure 14) with the size of the shown agglomerate being approximately 15 µm × 7 µm.

### 3.7. 3D Printing

**Figure 17:**
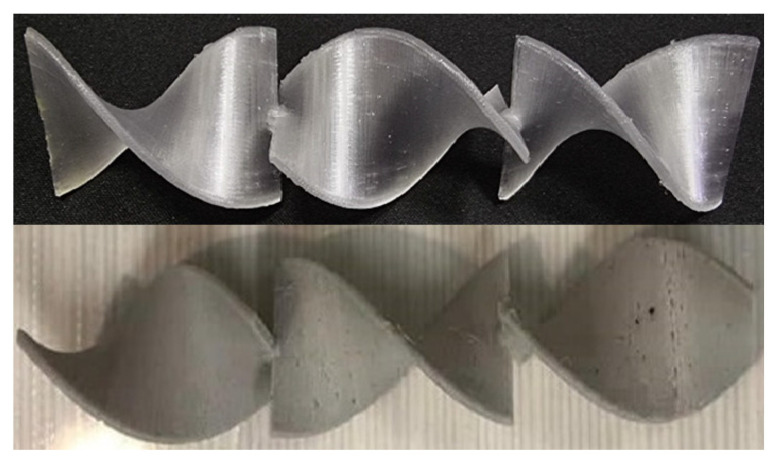
Three-dimensional-printed static mixers made of pure PET-G (**top**) and PET-G 6T 0.25C composite (**bottom**).

**Figure 18:**
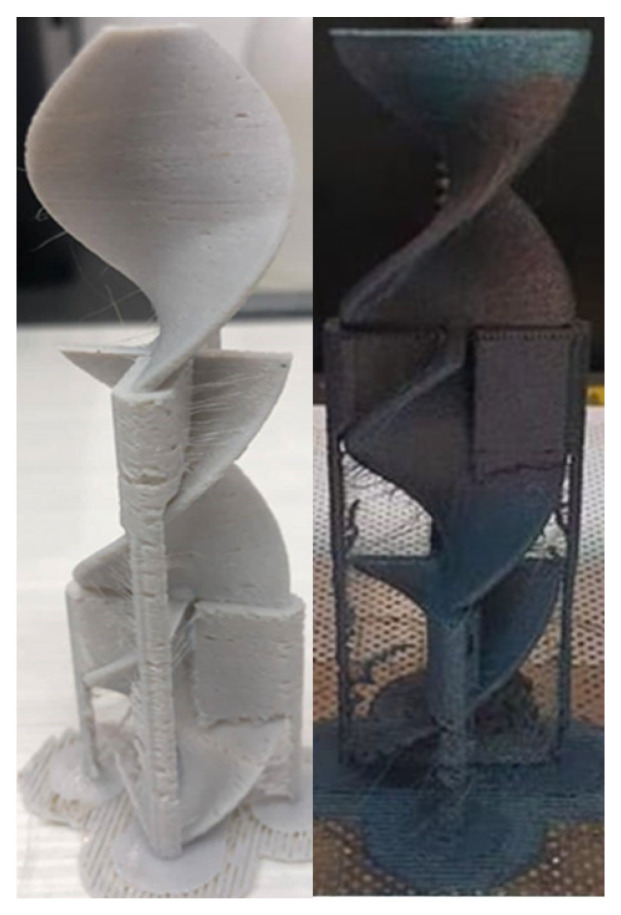
Three-dimensional-printed static mixers made of composites PET-G 6T 0C (**left**) and PET-G 1.5T 0.5C (**right**) before the removal of the support structures.

All of the prepared composites were successfully used for 3D printing. Some of the printed static mixers are shown in Figure 17 and Figure 18. In Figure 17, on the bottom static mixer 3D printed from the composite material, imperfections on the surface can be observed. Those imperfections are formed because of the clogging of the nozzle and/or unequal flow of the material due to the previously mentioned inhomogeneity of the composite materials. Additionally, imperfections on the surface can occur as unwanted marks after support structure removal. The surface containing said imperfections in this specific case can have a positive impact because of the specific-surface-area increase.

## 4. Conclusions

Functional filament composites of a PET-G polymer matrix with TiO_2_ and CNT fillers were successfully prepared using a Noztek Pro extruder. The diameter of the prepared filaments was within 5% deviation from 1.75 mm, as required for a 3D printer. All of the prepared composites were used for successful 3D printing using the Zortrax M200 3D printer, allowing all the planned static mixers to be successfully manufactured.

The thermal and mechanical properties of the composite materials were analyzed and compared to pure PET-G. MFR analysis indicated a decrease in viscosity of the composites compared to PET-G, reaching a plateau after 2 wt% fillers were added. Changes in the viscosity are not directly related to the addition of the fillers but to the degradation of the polymer matrix during processing. The glass transition temperature was determined using DSC analysis and no shift in the values was found, indicating that the addition of fillers cancels the influence of the previously mentioned polymer degradation on this property of the PET-G polymer. Additionally, the SEM micrographs confirmed good filler–matrix distribution and adhesion. Thermal decomposition temperature was determined using TGA analysis and it was determined that the addition of fillers will not influence the working range of the material at elevated temperatures. A significant influence of the fillers on the mechanical properties was found. The addition of the fillers impacted the overall strength and elongation of the material. Strength was significantly increased with the initial addition of the filler, but it began to decrease with the further increase in the filler content. Elongation at break values were inversely proportional to those of strength. All results were within the required values for the 3D printing of the static mixers and the final application in the tubular reactors for polluted-water purification.

## Figures and Tables

**Table 1:** Mass ratios of added fillers for all samples.

	Matrix	Filler Ratio	
		*w* (TiO_2_) [%]	*w* (CNT) [%]
PET-G	PET-G	0.00	0.00
PET-G 1.5T 0C	PET-G	1.50	0.00
PET-G 1.5T 0.25C	PET-G	1.50	0.25
PET-G 1.5T 0.5C	PET-G	1.50	0.50
PET-G 3T 0C	PET-G	3.00	0.00
PET-G 3T 0.25C	PET-G	3.00	0.25
PET-G 3T 0.5C	PET-G	3.00	0.50
PET-G 6T 0C	PET-G	6.00	0.00
PET-G 6T 0.25C	PET-G	6.00	0.25
PET-G 6T 0.5C	PET-G	6.00	0.50

**Table 2:** Defined temperature zones for the twin-screw extruder.

Sample	Temperature Zones [°C]		
	First	Second	Third
PET-G	190	200	210
PET-G 1.5T 0C	190	200	210
PET-G 1.5T 0.25C	190	200	210
PET-G 1.5T 0.5C	190	200	210
PET-G 3T 0C	190	200	210
PET-G 3T 0.25C	190	200	210
PET-G 3T 0.5C	190	200	210
PET-G 6T 0C	195	205	215
PET-G 6T 0.25C	195	205	215
PET-G 6T 0.5C	195	205	215

**Table 3:** MFR test results of PET-G and prepared composites.

Sample	MFR [g/10 min]
PET-G	28.4
PET-G 1.5T 0C	28.5
PET-G 1.5T 0.25C	31.7
PET-G 1.5T 0.5C	34.2
PET-G 3T 0C	22.9
PET-G 3T 0.25C	15.9
PET-G 3T 0.5C	33.0
PET-G 6T 0C	33.2
PET-G 6T 0.25C	33.0
PET-G 6T 0.5C	33.9

**Table 4:** DSC test results of PET-G and composite samples.

Sample	Glass Transition [°C]
PET-G	73
PET-G 3T 0C	75
PET-G 3T 0.5C	73
PET-G 6T 0C	74
PET-G 6T 0.25C	73
PET-G 6T 0.5C	75

**Table 5:** TGA test results of PET-G and composite samples.

Sample	Decomposition Onset Temperature [°C]
PET-G	447
PET-G 6T 0C	445
PET-G 6T 0.5C	443

**Table 6:** Tensile test results of PET-G and prepared composites.

	Tensile Strength, *σ*_M_ [N mm^−2^]	Strain at *σ*_M_, *ε*_M_ [%]	Stress at Break, *σ*_B_ [N mm^−2^]	Strain at *σ*_B_, *ε*_B_ [%]
PET-G	17.4	4.1	9.6	12.3
PET-G 1.5T 0C	50.1	6.8	50.1	6.8
PET-G 1.5T 0.25C	36.0	4.1	36.0	4.1
PET-G 1.5T 0.5C	29.2	3.3	29.2	3.3
PET-G 3T 0C	43.8	5.4	43.8	5.4
PET-G 3T 0.25C	38.9	4.7	38.9	4.7
PET-G 3T 0.5C	38.6	4.7	38.6	4.7
PET-G 6T 0C	40.6	4.1	40.6	4.1
PET-G 6T 0.25C	35.7	4.0	35.7	4.0
PET-G 6T 0.5C	31.6	3.4	31.6	3.4
